# Novel *ALMS1* mutations in Chinese patients with Alström syndrome

**Published:** 2013-09-07

**Authors:** Xiaofang Liang, Hui Li, Huajin Li, Fei Xu, Fangtian Dong, Ruifang Sui

**Affiliations:** Department of Ophthalmology, Peking Union Medical College Hospital, Peking Union Medical College and Chinese Academy of Medical Sciences

## Abstract

**Purpose:**

Alström syndrome (AS) is a rare monogenic autosomal recessively inherited disorder characterized by cone rod dystrophy and multiple organ dysfunction. Mutations in the Alström syndrome 1 (*ALMS1*) gene have been found to be causative for AS. The purpose of this study was to identify *ALMS1* mutations and to assess the clinical features of Chinese patients with AS.

**Methods:**

Detailed ocular and laboratory examinations were performed. Peripheral blood samples were collected from patients and their parents. Genomic DNA was extracted with a Qiagen kit. Exons and exon/intron junctions of *ALMS1* were amplified with polymerase chain reaction (PCR) and screened for mutations with Sanger sequencing. The results were compared with the *ALMS1* transcript to exclude polymorphisms and confirm pathogenic mutations.

**Results:**

Seven patients from five unrelated non-consanguineous families were diagnosed with AS. All patients had cone rod dystrophy with impaired visual acuity, photophobia, and nystagmus. Other clinical features, including sensorineural hearing loss, truncal obesity, insulin resistance, type 2 diabetes mellitus, renal and hepatic dysfunction, hyperlipidemia, hypothyroidism, mental retardation, acanthosis nigricans, and scoliosis, were present. Sequencing revealed two novel mutations, p.N3150Kfs2X and p.V3154Xfs, in patient 1; one novel mutation, p.N3672Ifs11X, and one previously reported nonsense mutation, p.R3703X, in patient 2; novel mutations p.S2479X and p.R3611Efs7X in patient 3; one novel homozygous mutation, p.S695X, in patients 4 and 5; and two novel mutations, p.H688HfsX and p.Q3147Qfs2X, in patients 6 and 7. These mutations were not present in 100 unrelated healthy Chinese control subjects. The patients’ parents were heterozygous carriers of the mutant allele.

**Conclusions:**

Seven Chinese patients with AS showed typical ophthalmic features and multiple organ dysfunction. Novel loss of function mutations in the *ALMS1* gene are the underlying genetic defects.

## Introduction

Alström syndrome (MIM 203800) is a rare autosomal recessively inherited disorder with a prevalence of less than 1 per million in the general population [1]. Patients develop progressive cone rod dystrophy leading to blindness, sensorineural hearing loss (SNHL), early onset truncal obesity, hyperinsulinemia and insulin resistance, type 2 diabetes mellitus, hypertriglyceridemia, short stature in adulthood, dilated cardiomyopathy, recurrent pulmonary infections, hypogonadism, and hepatic and renal dysfunction. Fibrosis of unknown etiology develops in multiple organs [2]. AS is caused by mutations in the Alström syndrome 1 (*ALMS1*) gene, which locates on chromosome 2p13.1, spanning 23 exons and encoding a predicted 461.2 kDa protein of 4,169 amino acids (aa). *ALMS1* has been shown to be widely expressed and localized to the centrosomes and basal bodies of ciliated cells of tissues including the central nervous, photoreceptor, endocrine, cardiopulmonary, reproductive, and urological systems, suggesting roles in intracellular trafficking and ciliary function [3,4].

Pathogenic mutations of *ALMS1* have been identified in various ethnic populations [1-3], but only one nonsense homozygous mutation has been reported in a Chinese boy with AS [5]. Here we report clinical and novel molecular findings in seven Chinese patients from five different pedigrees with distinct features of AS.

## Methods

### Clinical evaluation

Patients with AS were identified at the Ophthalmic Genetics Clinic at Peking Union Medical College Hospital (PUMCH), Beijing, China. The diagnosis of AS was given according to the criteria defined by Marshall et al. previously [6]. Patients underwent ophthalmologic evaluations, including best-corrected visual acuity, slit-lamp biomicroscopy, dilated indirect ophthalmoscopy, and fundus photography. Retinal structure was examined with optical coherence tomography (Topcon, Tokyo, Japan). Electroretinograms (ERGs; RetiPort ERG system; Roland Consult, Wiesbaden, Germany) were performed using corneal “ERGjet” contact lens electrodes. The ERG protocol complied with the standards published by the International Society for Clinical Electrophysiology of Vision (ISCEV). Auditory examinations were conducted by otolaryngologists. Fasting venous blood samples were analyzed for glucose, lipid, lipoprotein, and hemogram levels, as well as renal and hepatic functions. The study protocol was approved by the Institutional Review Board of PUMCH and adhered to the tenets of the Declaration of Helsinki. All research subjects gave informed consent.

### Genetic analysis

Genomic DNA was isolated from peripheral blood using a QIAamp DNA Blood Midi Kit (Qiagen, Hilden, Germany) according to the manufacturer’s protocol. Polymerase chain reactions were designed to amplify *ALMS1* exons and splice-site sequences. Primers were synthesized according to sequences designed previously [7]. The final volume of 50 μl contained 40 ng genomic DNA, 10 pmol of each primer, and 25 μl 2×Taq PCR Master Mix (Biomed Technologies, Beijing, China). DNA amplifications were performed with denaturing at 94 °C for 5 min, followed by 30 cycles of a denaturing step at 94 °C, an annealing step at 60 °C, and an extension step at 72 °C, each for 30 s. A final extension step at 72 °C was performed for 7 min. Amplification products were electrophoresed on 1.5% agarose gels. The sequences were analyzed using the Blat tool compared with the *ALMS1* transcript (GenBank Accession No. NM_015120). The variant sequence was confirmed in the patients’ parents for cosegregation. To confirm the variants were not nonpathogenic polymorphisms, direct sequencing was used in 100 unrelated healthy Chinese control subjects.

## Results

### Genetic evaluation

**Figure 1:**
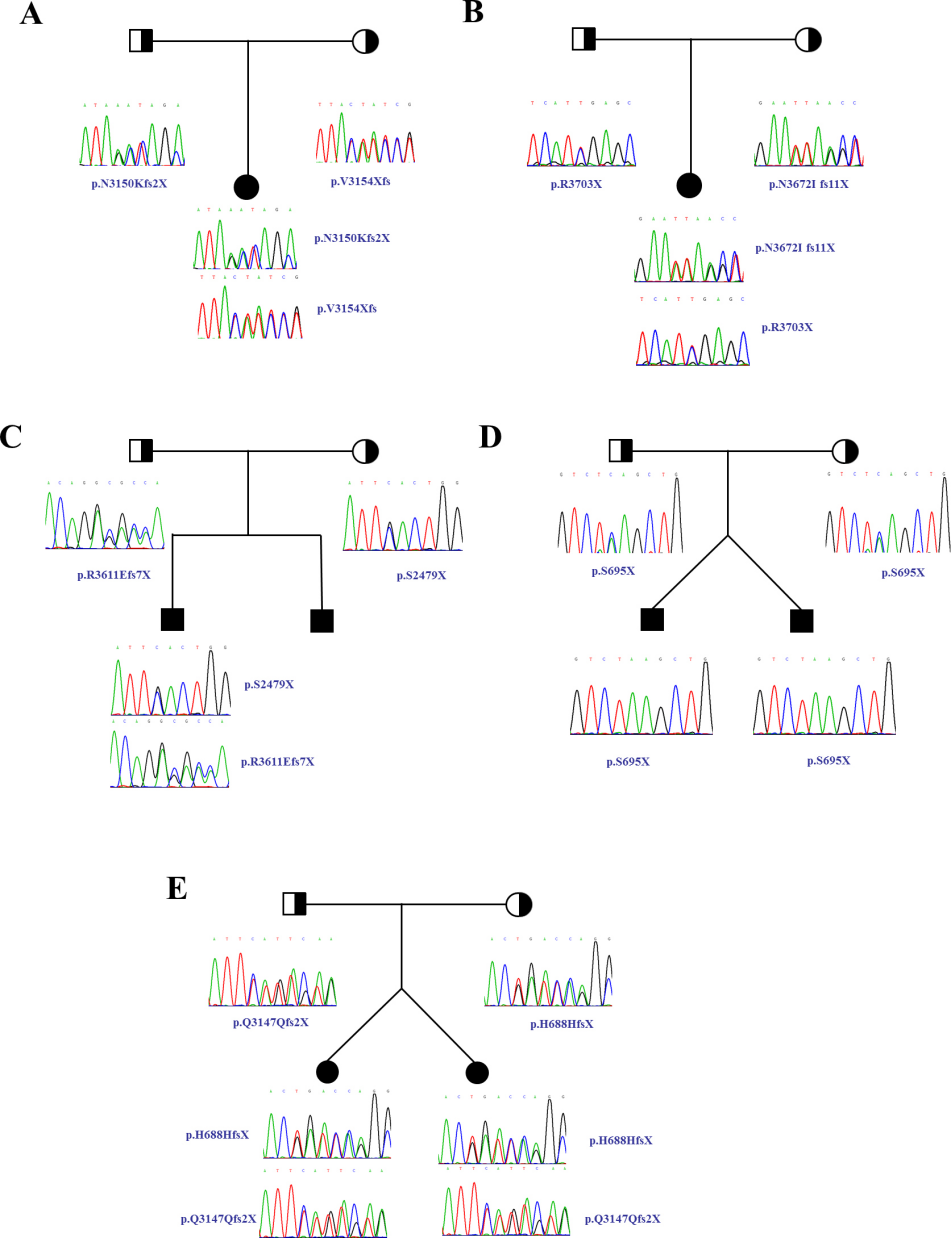
Pedigree and sequence analysis of five families. Patients are all compound heterozygous in the loci. Parents were heterozygous carriers of the mutant allele. **A**: Patient 1’s family. Patient 1 carried two mutations, p.N3150Kfs2X (c.9448insA) and p.V3154Xfs (C.9460delG); father, p.N3150Kfs2X; mother p.V3154Xfs. **B**: Patient 2’s family. Patient 2 carried two mutations, p.N3672Ifs11X (c.11015delA) and P.R3703X (c.11107C>T); father, P.R3703X; mother p.N3672Ifs11X. **C**: Patient 3′s family. Patient 3 carried two mutations, p.S2479X (c.7436C>G) and p.R3611Efs7X (c.10883insG); father, p.R3611Efs7X; mother, p.S2479X. **D**: Patients 4 and 5′s family. Patients 4 and 5 carried a homozygous mutation, p.S695X (c.2084C>A), father, p.S695X; mother, p.S695X. E: Patients 6 and 7’s family. Patients 6 and 7 carried two mutations, p.H688HfsX (c.2064delT) and p.Q3147Qfs2X (c.9441_9442insAATA); father, p.Q3147Qfs2X; mother, p.H688HfsX. Squares indicate men; circles, women; black, patient; half black, mutant allele carrier.

All of the detected mutations cause premature termination of the protein. The patients’ parents were heterozygous carriers of the mutant allele. Sequencing in patient 1 revealed two novel mutations in exon 10, including a 1-bp insertion, c.9448insA (p.N3150Kfs2X), and a 1-bp deletion, c.9460delG (p.V3154Xfs; Figure 1A). Sequencing in patient 2 identified a novel 1-bp deletion, c.11015delA (p.N3672Ifs11X), and a previously reported nonsense mutation, c.11107C>T (P.R3703X), in exon 16 [5] (Figure 1B). Sequencing in patient 3 revealed a novel nonsense mutation, c.7436C>G (p.S2479X), in exon 8 and a novel 1-bp insertion mutation, c.10883insG (p.R3611Efs7X), in exon 16 (Figure 1C). Sequencing in twin brothers, patients 4 and 5, revealed a novel homozygous mutation, c.2084C>A (p.S695X), in exon 8 (Figure 1D). Sequencing in twin sisters, patients 6 and 7, revealed a novel 1-bp deletion mutation, c.2064delT (p.H688HfsX), in exon 8 and a novel 4-bp novel insertion mutation, c.9441_9442insAATA (p.Q3147Qfs2X), in exon 10 (Figure 1E). These mutations were not present in 100 unrelated healthy Chinese control subjects.

### Clinical evaluation

A total of seven patients (four women and three men) from five unrelated non-consanguineous families were recruited for this study. Patient age ranged from 5 to 14 years old. Patients 4 and 5 are twin brothers, and patients 6 and 7 are twin sisters. Some had been previously diagnosed with early onset severe retinitis pigmentosa (patient 1), Joubert syndrome (patient 1), Leber congenital amaurosis (patients 3, 4, and 5), and Bardet-Biedl syndrome (BBS; patients 2 and 3). A summary of the clinical evaluation of the seven patients is shown in Table 1.

**Table 1:** Clinical information and phenotype of 7 patients.

Variables	Patient 1	Patient 2	Patient 3	Patient 4 and 5	Patient 6 and 7
Mutant allele 1	c.9448 ins A p.N3150Kfs2X	c.11015 del A p.N3672I fs11X	c.7436C>G p.S2479X	c.2084C>A p.S695X	c.2064delT p.H688HfsX
Mutant allele 2	c.9460 del G p.V3154Xfs	c.11107C>T P.R3703X	c.10883insG p.R3611Efs7X	c.2084C>A p.S695X	c.9441_9442insAATA p.Q3147Qfs2X
Mutant exon	10	16	8 and 16	8	8 and 10
Gender	female	female	male	male	female
Age (year)	14	13	5	13	7
BMI (kg/m^2^)	24	26.7	24	24.3	25.1
BCVA	LP	20/200	CF	20/200 and 10/200	10/200
Cone rod dystrophy	Y	Y	Y	Y	Y
T2DM	Y	Y	N	Y	N
SNHL	Y	Y	N	N	N
Hepatic dysfunction	Y	N	Y	Y	Y
Renal dysfunction	Y	Y	N	Y	Y
Hypothyoid dysfunction	Y	N	N	N	N
Hypogonadism	N	N	Y	N	N
Acanthosis nigricans	Y	Y	N	Y	N
Mental retardation	Y	Y	Y	N	N
Scoliosis	Y	N	N	N	N
Epilepsy	Y	N	N	N	N

Y refers to patient was examined and phenotype was present; N, not observed.

**Figure 2:**
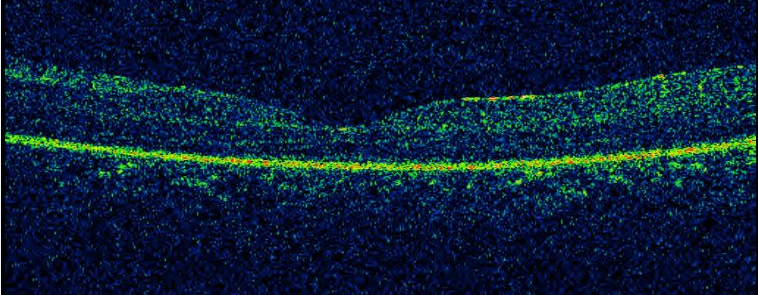
Optical coherence tomography for patient 2. Optical coherence tomography (OCT) showed a thinned retinal pigment epithelium and a photoreceptor layer.

**Figure 3:**
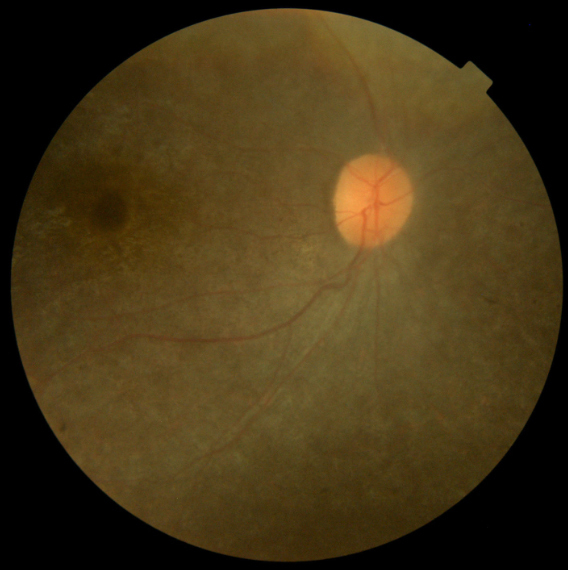
Fundus photograph of patient 2. The fundus showed salt and pepper pigmentation variation and attenuation of the retinal vessels.

**Figure 4:**
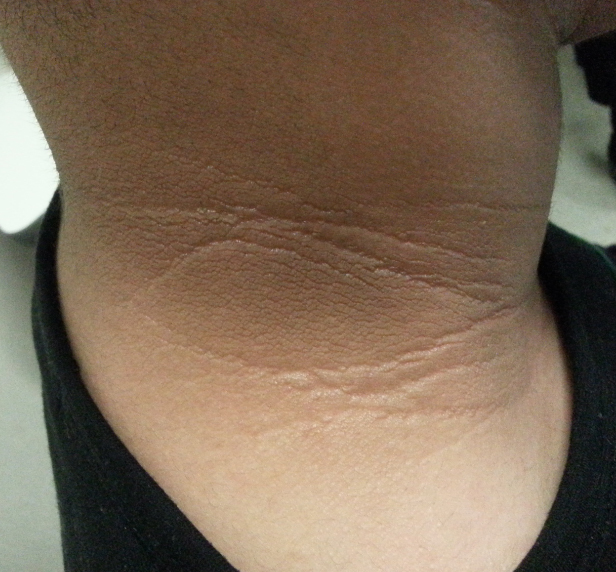
Acanthosis nigricans of patient 4’s neck..

Nystagmus and photophobia were present in all patients and as the first clinical manifestation. Vision loss progressed rapidly in the patients’ early childhood, and the patients’ best-corrected visual acuity ranged from LP to 20/200 when they presented to our clinic. ERG was unrecordable, and optical coherence tomography showed thinned retinal pigment epithelium and inner segment/outer segment complex in all patients (Figure 2). The fundus showed different variations, including sallow optic disc (patient 1), gold foil appearance of the macula (patients 2 and 3; Figure 3), salt and pepper pigmentation changes (patients 1 and 2), greyish pigments (patients 4 and 5), and attenuation of retinal vessels (all patients). Other clinical features including truncal obesity (all patients), type 2 diabetes mellitus (patients 1, 2, 4, and 5), SNHL (patients 1 and 2), hepatic dysfunction and renal dysfunction (all patients except patient 2), hypothyroidism (patient 1), hyperlipidemia (patients 1 and 2), hypogonadism (patient 3), acanthosis nigricans (patients 1, 2, 4, and 5; Figure 4), mental retardation (patients 1, 2, and 3), scoliosis (patient 1), and epilepsy (patient 1) were also observed.

## Discussion

Alström syndrome was first reported in 1959 [8], but has been recognized as a monogenic ciliopathy only in the past 5 years [9]. The diagnosis of AS is based on cardinal clinical features that emerge throughout infancy, childhood, and adulthood. All seven patients in this study meet the diagnostic criteria for AS [6]. Although clinical variability was observed among these subjects, the first sign of AS was nystagmus and photophobia caused by cone rod dystrophy. Other systemic clinical characteristics that appear later include truncal obesity, SNHL, hepatic and renal dysfunction, hypothyroid dysfunction, acanthosis nigricans, and so on. AS bears a close clinical resemblance to BBS, a known multigenic ciliopathy also characterized by childhood obesity and retinal dystrophy. Differentiating between BBS and AS in diagnosis can be challenging, especially in early infancy, when signs of AS have not yet been fully expressed. If only the eye exhibits abnormality in the first few years of life, AS may also be misdiagnosed as early onset severe retinitis pigmentosa, Leber congenital amaurosis, or achromatopsia.

*ALMS1* is the only gene in which mutations are known to cause AS. *ALMS1* sequencing can be helpful in providing the correct diagnosis for patients such as those of this study. Appropriate genetic counseling and medical follow-up may be performed based on genetic data. By adding seven frameshift mutations and one nonsense mutation, this study increases the total number (109 to 117) of reported *ALMS1* disease-causing variants. Novel mutations account for 90% of all mutations we detected. Interestingly, in our study, the mutant loci in patient 1 were close together; we detected only one frameshift mutation in the sequencing data. After analyzing the sequence of her parents, we confirmed the second frameshift mutation in the reverse sequence of patient 1.

Previous studies have showed that the majority of *ALMS1* mutations are nonsense and frameshift variations (insertions or deletions) that are predicted to cause premature protein truncation [3,10-14]. Most disease-causing mutations described thus far are located in the coding regions of exons 8, 10, and 16, with mutations in exon 16 accounting for 36% of the total mutations in AS [15]. The most common *ALMS1* mutation is c.10775delC, which is observed in 50% of reported English kindred [1]. A screening strategy that first targets the regions of exons 16, 10, and 8 and then detects the coding region of other exons should be successfully used. Most reports on AS are from Caucasian kindred. This may be due to developed countries having more readily available health care access compared to developing countries. Patients with AS who shared several features with the common metabolic syndrome were perhaps misdiagnosed if doctors did not provide a comprehensive evaluation. Haplotype sharing has revealed the possibility of separate founder effects in English and Turkish populations [1], which may be the reason that novel mutations account for up to 90% of mutations in our Chinese patients.

AS exhibits a great degree of phenotypic variations in the onset and severity of the phenotypes. AS also displays genetic heterogeneity, yet significant genotype–phenotype correlations have not been found. Mutation c.11107C>T (p.R3703X), first identified in a consanguineous family in the United Kingdom [9], was also detected in patient 2, but the clinical features in each case were different, as the previously reported patient showed signs of dilated cardiomyopathy. These different clinical features suggest that there is potential for allelic effects on disease manifestation, as well as regarding genetic background and environmental modification.

In this article, we report a comprehensive molecular and clinical study of seven Chinese patients with AS, the largest Chinese patient cohort studied to date. Misdiagnosis of AS may be high in developing countries such as China. Therefore, genetic testing is important for suspected AS cases. Early medical interventions in progressive multiorgan dysfunction are crucial for better disease management. The mutations described here may eventually provide further insight into the function of the *ALMS1* protein and contribute to the understanding of the phenotypic variety of AS.

## References

[1] Marshall JD, Hinman EG, Collin GB, Beck S, Cerqueira R, Maffei P, Milan G, Zhang W, Wilson DI, Hearn T, Tavares P, Vettor R, Veronese C, Martin M, So WV, Nishina PM, Naggert JK (2007). Spectrum of ALMS1 variants and evaluation of genotype-phenotype correlations in Alström syndrome.. Hum Mutat.

[2] Marshall JD, Bronson RT, Collin GB, Nordstrom AD, Maffei P, Paisey RB, Carey C, Macdermott S, Russell-Eggitt I, Shea SE, Davis J, Beck S, Shatirishvili G, Mihai CM, Hoeltzenbein M, Pozzan GB, Hopkinson I, Sicolo N, Naggert JK, Nishina PM (2005). New Alstrom syndrome phenotypes based on the evaluation of 182 cases.. Arch Intern Med.

[3] Collin GB, Marshall JD, Ikeda A, So WV, Russell-Eggitt I, Maffei P, Beck S, Boerkoel CF, Sicolo N, Martin M, Nishina PM, Naggert JK (2002). Mutations in ALMS1 cause obesity, type 2 diabetes and neurosensory degeneration in Alström syndrome.. Nat Genet.

[4] Collin GB, Marshall JD, Boerkoel CF, Levin AV, Weksberg R, Greenberg J, Michaud JL, Naggert JK, Nishina PM (1999). Alstrom syndrome: further evidence for linkage to human chromosome 2p13.. Hum Genet.

[5] Liu L, Dong B, Chen X, Li J, Li Y (2009). Identification of a novel ALMS1 mutation in a Chinese family with Alström syndrome.. Eye (Lond).

[6] Marshall JD, Beck S, Maffei P, Naggert JK (2007). Alström syndrome.. Eur J Hum Genet.

[7] Joy T, Cao H, Black G, Malik R, Charlton-Menys V, Hegele RA, Durrington PN (2007). Alstrom syndrome (OMIM 203800): a case report and literature review.. Orphanet J Rare Dis.

[8] Alstrom CH, Hallgren B, Nilsson LB, Asander H (1959). Retinal degeneration combined with obesity, diabetes mellitus and neurogenous deafness: a specific syndrome (not hitherto described) distinct from the Laurence-Moon-Bardet–Biedl syndrome: aclinical, endocrinological and genetic examination based on a large pedigree.. Acta Psychiatr Neurol Scand, Suppl.

[9] Baker K, Beales PL (2009). Making sense of cilia in disease: the human ciliopathies.. Am J Med Genet C Semin Med Genet.

[10] Bond J, Flintoff K, Higgins J, Scott S, Bennet C, Parsons J, Mannon J, Jafri H, Rashid Y, Barrow M, Trembath R, Woodruff G, Rossa E, Lynch S, Sheilds J, Newbury-Ecob R, Falconer A, Holland P, Cockburn D, Karbani G, Malik S, Ahmed M, Roberts E, Taylor G, Woods CG (2005). The importance of seeking ALMS1 mutations in infants with dilated cardiomyopathy.. J Med Genet.

[11] Hearn T, Renforth GL, Spalluto C, Hanley NA, Piper K, Brickwood S, White C, Connolly V, Taylor JF, Russell-Eggitt I, Bonneau D, Walker M, Wilson DI (2002). Mutation of ALMS1, a large gene with a tandem repeat encoding 47 amino acids, causes Alstrom syndrome.. Nat Genet.

[12] Kinoshita T, Hanaki K, Kawashima Y, Nagaishi J, Hayashi A, Okada S, Murakami J, Nanba E, Tomonaga R, Kanzaki S (2003). A novel non-sense mutation in Alstrom syndrome: subcellular localization of its truncated protein.. Clin Pediatr Endocrinol.

[13] Titomanlio L, De Brasi D, Buoninconti A, Sperandeo MP, Pepe A, Andria G, Sebastio G (2004). Alstrom syndrome: intrafamilial phenotypic variability in sibs with a novel nonsense mutation of the ALMS1 gene.. Clin Genet.

[14] Minton JA, Owen KR, Ricketts CJ, Crabtree N, Shaikh G, Ehtisham S, Porter JR, Carey C, Hodge D, Paisey R, Walker M, Barrett TG (2006). Syndromic obesity and diabetes: changes in body composition with age and mutation analysis of ALMS1 in 12 United Kingdom kindreds with Alstrom syndrome.. J Clin Endocrinol Metab.

[15] Marshall JD, Maffei P, Collin GB, Naggert JK (2011). Alström syndrome: genetics and clinical overview.. Curr Genomics.

